# Knockdown of NR3C1 inhibits the proliferation and migration of clear cell renal cell carcinoma through activating endoplasmic reticulum stress–mitophagy

**DOI:** 10.1186/s12967-023-04560-2

**Published:** 2023-10-08

**Authors:** Minbo Yan, Jinhua Wang, Haojie Wang, Jun Zhou, Hao Qi, Yaser Naji, Liangyu Zhao, Yuxin Tang, Yingbo Dai

**Affiliations:** 1 Department of Urology, The Fifth Affiliated Hospital of Sun Yat-Sen University, Zhuhai, Guangdong China; 2 Guangdong Provincial Key Laboratory of Biomedical Imaging, The Fifth Affiliated Hospital, Sun Yat-Sen University, Zhuhai, Guangdong China

**Keywords:** NR3C1, Clear cell renal cell carcinoma, Endoplasmic reticulum stress, Mitophgay

## Abstract

**Background:**

Clear cell renal cell carcinoma (ccRCC) is closely associated with steroid hormones and their receptors affected by lipid metabolism. Recently, there has been growing interest in the carcinogenic role of NR3C1, the sole gene responsible for encoding glucocorticoid receptor. However, the specific role of NR3C1 in ccRCC remains unclear. The present study was thus developed to explore the underlying mechanism of NR3C1’s carcinogenic effects in ccRCC.

**Methods:**

Expression of NR3C1 was verified by various tumor databases and assessed using RT-qPCR and western blot. Stable transfected cell lines of ccRCC with NR3C1 knockdown were constructed, and a range of in vitro and in vivo experiments were performed to examine the effects of NR3C1 on ccRCC proliferation and migration. Transcriptomics and lipidomics sequencing were then conducted on ACHN cells, which were divided into control and sh-NR3C1 group. Finally, the sequencing results were validated using transmission electron microscopy, mitochondrial membrane potential assay, immunofluorescence co-localization, cell immunofluorescent staining, and Western blot. The rescue experiments were designed to investigate the relationship between endoplasmic reticulum stress (ER stress) and mitophagy in ccRCC cells after NR3C1 knockdown, as well as the regulation of their intrinsic signaling pathways.

**Results:**

The expression of NR3C1 in ccRCC cells and tissues was significantly elevated. The sh-NR3C1 group, which had lower levels of NR3C1, exhibited a lower proliferation and migration capacity of ccRCC than that of the control group (*P* < 0.05). Then, lipidomic and transcriptomic sequencing showed that lipid metabolism disorders, ER stress, and mitophagy genes were enriched in the sh-NR3C1 group. Finally, compared to the control group, ER stress and mitophagy were observed in the sh-NR3C1 group, while the expression of ATF6, CHOP, PINK1, and BNIP3 was also up-regulated (*P* < 0.05). Furthermore, Ceapin-A7, an inhibitor of ATF6, significantly down-regulated the expression of PINK1 and BNIP3 (*P* < 0.05), and significantly increased the proliferation and migration of ccRCC cells (*P* < 0.05).

**Conclusions:**

This study confirms that knockdown of NR3C1 activates ER stress and induces mitophagy through the ATF6-PINK1/BNIP3 pathway, resulting in reduced proliferation and migration of ccRCC. These findings indicate potential novel targets for clinical treatment of ccRCC.

**Graphical Abstract:**

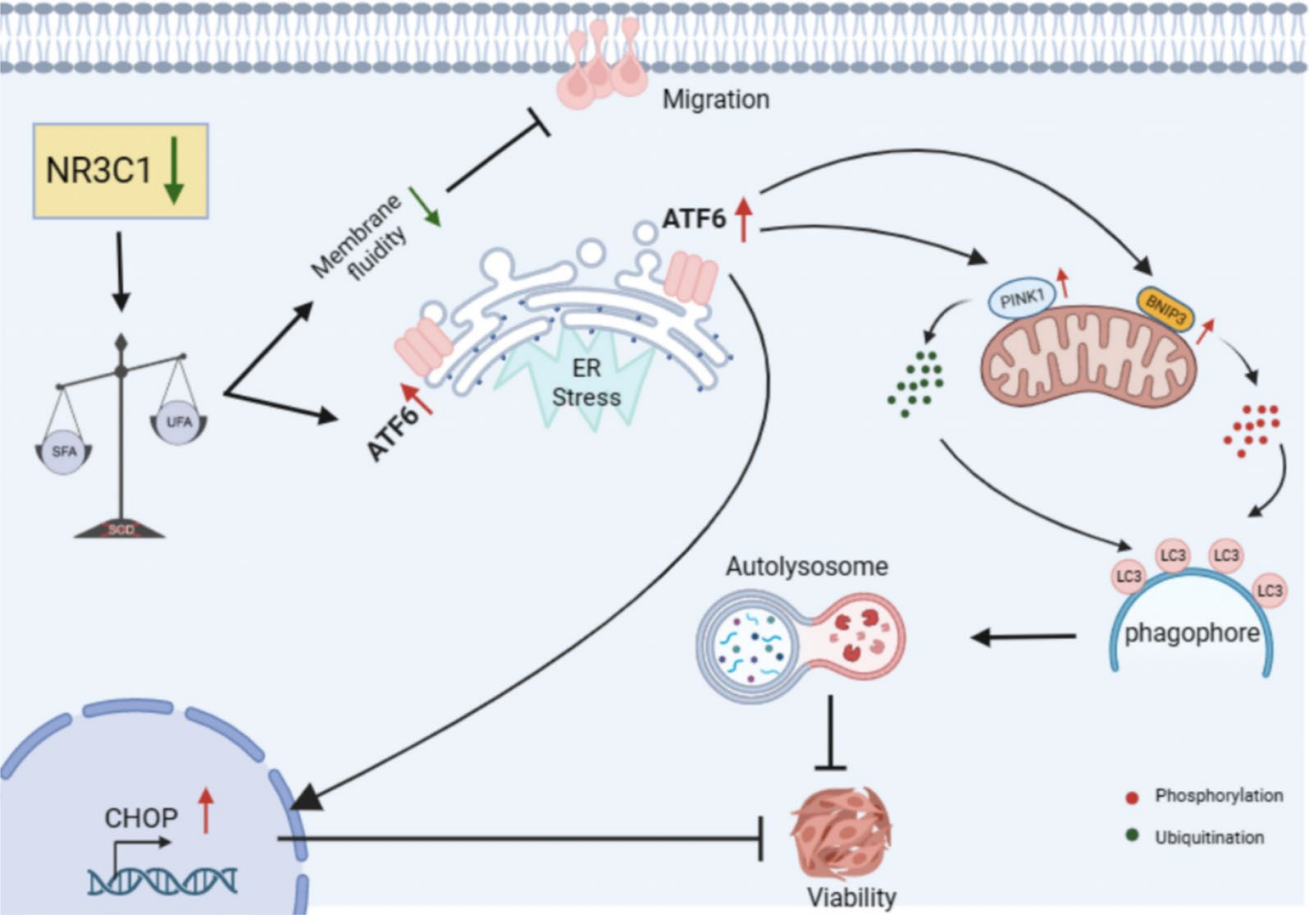

**Supplementary Information:**

The online version contains supplementary material available at 10.1186/s12967-023-04560-2.

## Introduction

Renal cancer is one of the most common solid malignancies of the genitourinary system. According to US cancer statistics, there are an estimated 81,800 newly diagnosed cases of renal cancer and approximately 14,890 attributed deaths in 2023 [1]. Clear cell renal cell carcinoma (ccRCC) is the most common type among all renal cancers, with its incidence increasing yearly [2]. Moreover, due to its silent nature, about 30% of patients with ccRCC are found to have metastasis at the time of initial diagnosis [3]. Thus, those patients have a low 5-year survival rate and poor prognosis [4]. Patients with metastatic ccRCC have very limited treatment modalities. That is because ccRCC are insensitive to chemoradiotherapy, and the overall efficacy of targeted therapy and immunotherapy still needs further improvement. Therefore, deeply exploring the mechanism of ccRCC and finding novel scientific and practical therapeutic strategies are still the primary focus of research at this stage.

The hypothesis that ccRCC is a hormone-dependent tumor was first proposed in 1978 [5, 6]. Since then, epidemiological, clinical, biochemical and genetic studies have confirmed the close relationship between multiple hormones and their receptor signaling pathways with ccRCC [7]. While the steroid receptor signaling pathway, such as the glucocorticoid receptor (GR), may be a novel target for anticancer therapy in ccRCC patients [8]. Nuclear receptor subfamily 3, group C, member 1 (NR3C1), the sole gene responsible for encoding GR, is located on chromosome 5q31.3, about 80 kb [9, 10]. Numerous studies have confirmed that NR3C1 can promote tumor proliferation, metastasis and drug resistance by up-regulating ROR-1 or NF-κB in various malignancies, such as triple-negative breast cancer, ovarian cancer, and urothelial cancer [11–14]. However, few studies have reported on the biological role of NR3C1 in ccRCC.

The endoplasmic reticulum (ER) is essential in monitoring cell proteins by degrading unfolded or misfolded proteins. When cells are stimulated by various internal and external factors such as hypoxia, oxidative stress, and carbohydrate or lipid metabolism disorder, the ER will undergo morphological changes and functional imbalances. This leads to the failure of normal formation of disulfide bonds or protein mutations, resulting in a large number of unfolded or misfolded proteins accumulating in the ER, and inducing endoplasmic reticulum stress (ER stress) [15].

In 2005, Lemasters [16] first proposed the concept of mitophagy, that is, when mitochondria are damaged by internal and external factors, mitochondrial receptor proteins form autophagic membranes by interacting with LC3/GABARAP family members, and these autophagic membranes specifically recognize and encapsulate damaged mitochondria, which are then degraded and cleared by binding to lysosomes. Normally, ER stress and mitophagy are extremely important for maintaining cellular homeostasis. However, if cells are subjected to prolonged and intense stimulation, excessive activation of ER stress and mitophagy can lead to self-degradation of numerous organelles or accumulation of toxic substances, ultimately resulting in cell death [17]. Currently, the role of ER stress and mitophagy in malignancies has become the primary focus of basic research.

This study was intrigued by the functional role resulting from the elevated expression of NR3C1 in ccRCC. We discovered that the internal mechanism of NR3C1 in ccRCC is different from other malignancies. Herein, we provide the first evidence showing that knockdown of NR3C1 can inhibit the proliferation and migration of ccRCC by activating the ER stress and mitophagy.

## Materials and methods

### ccRCC tissues

Fresh tumor tissues (T) and peritumoral tissues (P) were collected from 10 patients pathologically diagnosed with ccRCC in the urology department of the Fifth Affiliated Hospital of Sun Yat-sen University from March 2022 to October 2022. The peritumoral tissue was required to be more than 1 cm away from the tumor border. All collected specimens were immediately stored in liquid nitrogen container. In this study, all patients were fully informed of the research content and purpose, and signed the “Surgery Informed Consent Form” and “Clinical Biological Resources Collection Informed Consent Form”. The acquisition of all human samples was approved by the Medical Ethics Committee of the Fifth Affiliated Hospital of Sun Yat-sen University (2021-L066-1). Clinicopathological characteristics of ccRCC patients are shown in Additional file 2: Table S1.

### Cell lines and cell culture

The human normal cell line HK-2 (#CL-0109) was purchased from Procell life science & technology co.ltd (Wuhan, China). The human cancer cell lines 786-O, ACHN, A-498 and UMRC6 were purchased from the American Type Culture Collection (ATCC). HK-2, A-498 and UMRC6 cells were cultured in Dulbecco’s Modified Eagle’s High Glucose Medium (4.5 g/L) (DMEM, Gibco); while 786-O and ACHN cells were cultured in Roswell Park Memorial Institute 1640 Medium (RPMI-1640, Gibco). The culture medium was supplemented with 10% fetal bovine serum (FBS) and 1% penicillin–streptomycin mixture. All cell lines were confirmed to be free of mycoplasma contamination prior to use and were incubated at 37 °C in a humidified atmosphere containing 5% CO_2_.

### Antibodies and regents

Antibodies against NR3C1(#A2164), ATF6(#A0202), p-eIF2α(#AP0692), PINK1(#A7131), HRP Goat Anti-Mouse IgG(#AS003), HRP Goat Anti-Rabbit IgG(#AS014) were purchased from Abclonal. Antibodies against β-actin(#81115), p-IRE1(#27528), PERK (#20582), CHOP (#15204) were purchased from Proteintech. Antibodies against LC3B (E5Q2K) (#83506), P62 (E7M1A) (#16177) were purchased from Cell Signaling Technology. Antibody against BNIP3 (#ab109362) was purchased from Abcam.

Dexamethasone sodium phosphate injection (#20220855) was purchased from Sancai Shiqi Pharmaceutical Co.Ltd (Zhongshan, China). Ceapin-A7 (#E1099) was purchased from Selleck Chemicals. LV-NR3C1-RNAi (#16863-1) was purchased from Genechem (Shanghai, China).

### RT-qPCR

Total RNA were extracted using E.Z.N.A HP Total RNA Kit I (Omega Bio-tek) and stored at – 80 ℃ after testing for concentration and purity. The HiScript III RT SuperMix for qPCR kit and ChamQ Universal SYBR qPCR Master Mix kit (Vazyme, Nanjing, China) were used for cDNA reverse transcription and PCR reaction. The total reaction volume was 20 μL which included 10 μL ChamQ Universal SYBR qPCR Master Mix(2 ×); 1 μL cDNA; 0.8 μL of forward and reverse primers; and sterile purified water. The amplification conditions were as follows: 95 ℃ for 30 s, 95 ℃ for 5 s, 60 ℃ for 30 s, and 40 cycles. The relative mRNA expression levels were determined by the 2− ΔΔCt method and the experiment was repeated three times.

The primer sequences were: NR3C1 forward primer–ACAGCATCCCTTTCTCAAC AG; NR3C1 reverse primer–AGATCCTTGGCACCTATTCCAAT; β-actin forward primer–GCATCGTCACCAACT GGGAC; β-actin reverse primer–ACCTGGCCGTCAG GCAGCTC.

### Western blot

Cell and tissue proteins were extracted using conventional methods, and protein concentrations were determined using BCA protein concentration kit (Beyotime, Shanghai, China). Equal amounts of protein were added to each well based on sample concentration, with a marker dosage of 5 μL. Electrophoresis was performed on a 4–12% gradient precast gel at a constant voltage of 120 V. The PVDF film was cut as needed and immersed in methanol for 5 min. Then the proteins were transferred to the PVDF film using a constant current of 350 mA. The PVDF film was then washed three times with TBST for 5 min each, followed by blocking with skim milk powder for 1 h at room temperature. Primary antibodies were incubated overnight at 4 °C and eluted the next day. Secondary antibodies were added for 1 h at room temperature and washed three times with TBST. Finally, 0.25 mL of each luminescent substrate solution and enhancer solution were taken for exposure. Images were developed using an Invitrogen iBright FL1500 gel imaging instrument and analyzed using Image J software. Primary antibodies were diluted to 1:1000 and secondary antibodies were diluted to 1:5000.

### Lentivirus cell transfection

Lentivirus cell transfection experiments were performed based on the Lentivirus Operation Manual (Genechem, Shanghai, China). The cell suspension was prepared with a density of 3–5 × 10^4^ cells/mL and seeded in a 6-well plate. LV-NR3C1-RNAi and HiTransG A infection reagent were added following the instructions. The cells were cultured for 48 h, and the medium was regularly replaced. Approximately 72 h after infection, the infection efficiency was observed using a fluorescence microscope. Stably transfected cell lines were screened with 1 μg/mL of puromycin. Transfection efficiency was evaluated by Western blot.

### Cell counting kit-8 (CCK-8) assay

Cell counting kit-8 (#CK04-50000 T) was purchased from Dojindo laboratorise (Japan). Briefy, ccRCC cells were seeded into 96-well plates at a density of 2 × 10^3^ cells in 100 μL media per well and cultured until the cell growth density reached approximately 80%. After adding 10 μL of CCK-8 solution, the cells were further incubated for 2 h away from light. A microplate reader was used to detect the OD value of each well at a wavelength of 450 nm. Cell viability was presented as a percentage of each concentration relative to control.

### EdU assay

According to the grouping design, GC group cells were pretreated with 1000 nM GC for 24 h. Then, all cells were seeded on a 96-well plate with a density of 2 × 10^4^/well and cultured until reaching the logarithmic growth phase. The following solutions were added sequentially: 100 μL 50 μM EdU solution (incubated for 2 h); 50 μL cell fixation solution containing 4% paraformaldehyde (incubated for 30 min); 50 μL 2 mg/mL glycine (incubated for 5 min); 100 μL 0.5% TritonX-100 in PBS (incubated for 10 min); 100 μL 1 × Apollo^®^ staining reaction solution (incubate in the dark for 30 min); 100 μL 0.5%TritonX-100 in PBS (incubated for 10 min); and 100 μL 1 × Hoechst staining reaction solution (100:1, incubate in the dark for 30 min). Finally, images were acquired from randomly selected fields under an inverted fluorescence microscope. The percentage of Edu-positive cells was calculated using Image J software. The Cell-Light EdU apollo567 in vitro kit (#C10310-1) were purchased from Ribobio (Guangzhou, China), and the Hoechst 33342 stain solution (#C0031) were purchased from Solarbio (Beijing, China).

### Colony formation assay

Cell suspensions with a density of 500 cells/mL were prepared and seeded in 6-well plates for 2 weeks, with medium changed every 3 days. The culture medium with or without GC was added according to the grouping design. Then the cells were washed three times with PBS, fixed with 1 mL of methanol for 15 min, and stained with 2 mL of 0.1% crystal violet for 1 h. Finally, the culture plates were dried by inverting on paper, and the colony formation was photographed and counted using Image J software. Gentian violet crystal violet staining solution (0.1%) (#BL802A) was purchased from Biosharp (Hefei, China).

### Wound healing assay

According to the grouping design, GC group cells were pre-treated with 1000 nM GC for 24 h. Then, all cells were seeded in 6-well plates at a density of 5 × 10^5^ cells/well for 24 h and wounded using a sterilized pipet tip to make a straight scratch. After that, the 6-well plates were incubated in an IncuCyte incubator (Essen bioscience, USA) and photographed every hour. Five fields were randomly selected to calculate the percentage of the disappearance of the wound surface area.

### Transwell assay

According to the grouping design, GC group cells were pretreated with 1000 nM GC for 24 h. Then, the cells were resuspended in serum-free medium. 600 μL of medium was added to the bottom chamber of a 24-well plate, while 100 μL of cell suspension at a density of 5 × 10^5^ cells/mL was seeded into the top chamber. After incubating at 37 ℃ for 12 h, the chambers were fixed in 4% paraformaldehyde for 30 min and stained with 0.1% crystal violet for 20 min. Cells in the bottom chambers were counted using an inverted phase-contrast microscope, with at least five randomly selected felds quantifed. The size and number of colonies were imaged and quantified with Image J software. Falcon^®^ Cell Culture Inserts (#353097) were purchased from Corning. Gentian violet crystal violet staining solution (0.1%) (#BL802A) was purchased from Biosharp (Hefei, China).

### Tumor formation in nude mice

Male BALB/C nude mice aged 6–8 weeks and weighing approximately 20 g were purchased from the Guangdong Provincial Medical Laboratory Animal Center. All animals were housed under pathogen-free conditions in the Guangdong Key Laboratory of Biomedical Imaging. Animal experiments were performed following the Guide for the Care and Use of Laboratory Animals, a publication of the National Institutes of Health. The animal study protocol was approved by the Animal Ethics Committee of the Fifth Affiliated Hospital of Sun Yat-sen University (00279). The specific steps were as follows:

The nude mice were divided into a control group and a sh-NR3C1 group, each seeded with ACHN cells at a density of 1 × 10^6^ cells/mL. The inoculation site was the upper middle inguinal subcutaneous region of nude mice, and the inoculation volume was 0.2 mL. Cells were individually seeded on different sides to distinguish between the two groups. Local invasive tumors could be reached approximately 30 days after inoculation, and the longest and shortest tumor diameters were measured using a vernier caliper, which were recorded every 3 days, and the volume was calculated using the formula V = 0.52ab^2^ (a = long axis; b = short axis). After continued feeding for about 3 weeks, nude mice were euthanized after isoflurane inhalation anesthesia, and the tumors were dissected intact and weighed.

### Mitochondrial membrane potential assay

The mitochondrial membrane potential assay kit with JC-1(#C2006) was purchased from Beyotime Biotechnology (Shanghai, China). The JC-1 staining working solution was prepared according to the kit instructions. Cells from the positive control group were treated with culture medium containing 10 µM CCCP for 20 min. To all cells, 0.5 mL of medium and 0.5 mL of JC-1 stained working solution were added and incubated at 37 ℃ for 20 min. For detecting the JC-1 monomer, the excitation light and emission light were set to 490 nm and 530 nm. For detecting the JC-1 polymer, the excitation light and emission light were set to 525 nm and 590 nm, respectively. The fluorescence intensity and the green-to-red (G/R) fluorescence ratio were quantified using Zen 2.3.

### Immunofluorescence co-localization

Cells were seeded onto confocal dishes at a density of 3 × 10^4^ cells/dish. Mito-Tracker Green (500 nM) and Lyso-Tracker Red (100 nM) were added successively, and the dishes were wrapped with tin foil and incubated for 30 min. The staining working solution was removed, and the dishes were washed twice with PBS before complete medium was added. Images were randomly acquired using a laser confocal microscope. Lyso-Tracker-Red (#L7528) was purchased from ThermoFisher. Mito-Tracker-Green (#9074) was purchased from Cell Signaling Technology.

### Transmission electron microscopy (TEM)

The cells were scraped from a 10 cm culture dish and transferred into centrifuge tubes. The cells were then centrifuged at 1500 rpm for 10 min and washed three times with PBS. Pellets were fixed in 2.5% electron microscopy-specific glutaraldehyde at 4 °C overnight. Following this, the cells were protected with dry ice and sent to Yanqu information technology Co. Ltd (Hangzhou, China) for transmission electron microscopy.

### Immunofluorescent staining

The cells were seeded onto 6-well plates at a density of 5 × 10^5^ cells/well and cultured for 24 h. After washing with PBS, the cells were fixed with 4% paraformaldehyde for 20 min. Then, the cells were treated with 0.4% Tryton X-100 for 20 min and blocked with 5% goat serum (Solarbio, Beijing, China) in PBS containing 1% BSA for 1 h. The LC3B antibody (1:200) was added and incubated overnight at 4℃. Following this, the cells were washed and stained with a secondary antibody (1:1000) for 1 h before being counterstained with DAPI glycerol mounting solution (1:400) for 5 min at room temperature. Finally, the cells were dried at room temperature, the anti-fluorescence quenching sealing agent was added, and the cover slides were placed. Fields were randomly selected and photographed under a fluorescence microscope.

### RNA sequencing

RNA sequencing analysis was performed by BGI Genomics Co. Ltd. (Shenzhen, China). Total RNA was extracted from each sample, and 2 µg of RNA from each sample was used to construct the sequencing cDNA library with a size range of 300–400 bp. The library quality was inspected using an Agilent 2100 Bioanalyzer (Agilent, USA). High-throughput dual-end sequencing of different libraries was performed using Illumina PE150 (Illumina, USA) based on the effective concentration and target data volume requirements. Finally, a differential gene expression analysis was conducted between groups, and Gene Ontology (GO) and Kyoto Encyclopedia of Genes and Genomes (KEGG) were used for classification and enrichment analysis of differentially expressed genes (DEGs).

### Lipidomics sequencing

Lipidomics sequencing analysis was performed by BGI Genomics Co. Ltd. (Shenzhen, China). Firstly, lipid metabolites were extracted, and quality control (QC) samples were prepared. Waters 2777C UPLC (Waters, USA) and Q Exactive HF High-resolution mass spectrometer (Thermo Fisher Scientific, USA) were used for the separation and detection of lipid metabolites. Data were then imported into LipidSearch v.4.1 software (Thermo Fisher Scientific, USA) for mass spectrometry analysis, and the lipid molecular identification results and quantitative results were obtained. Finally, the above data were used for data quality control, global metabolite analysis, screening of differentially expressed lipids between groups and unsaturation analysis of lipid molecules.

### Statistical analysis

All data were analyzed statistically using GraphPad Prism 8 and presented as mean ± standard deviation (Mean ± SD). All experiments were conducted three times, and the data represent the average of three independent experiments. The Student's t-test and one-way ANOVA were used to compare the differences between groups, with a *P*-value < 0.05 considered statistically significant. Statistical significance was indicated by asterisks as follows: *P < 0.05, **P < 0.01, ***P < 0.001, ****P < 0.0001.

## Results

### The expression of NR3C1 is elevated in ccRCC

**Fig.1:**
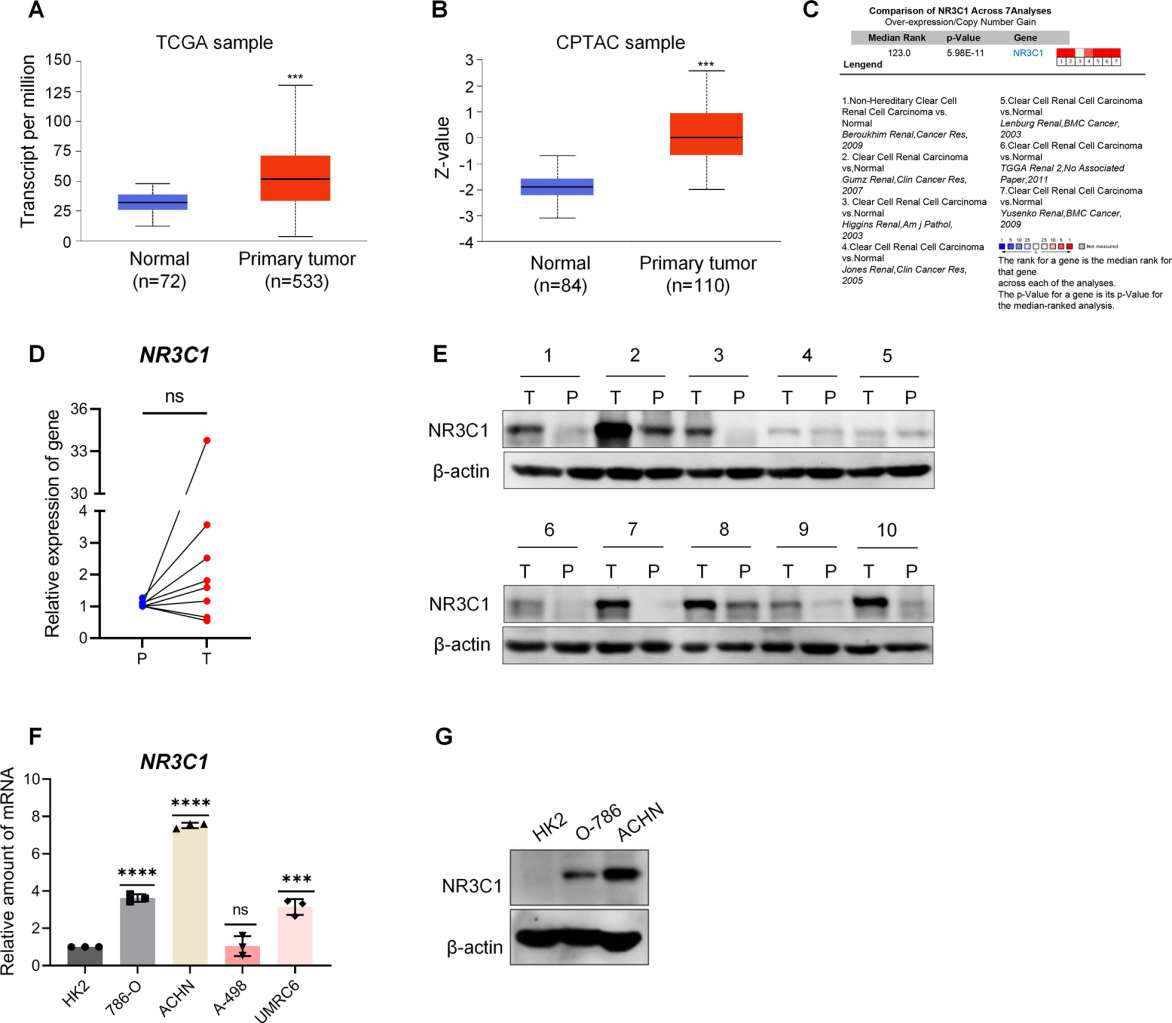
The expression of NR3C1 is elevated in ccRCC. **A** Expression of NR3C1 mRNA in ccRCC (n = 533) and adjacent normal (n = 72) tissues in the TCGA-KIRC cohort. **B** Expression of NR3C1 protein in ccRCC (n = 110) and adjacent normal (n = 84) tissues in the CPTAC database.** C** Meta-analysis of NR3C1 expression differences across 7 analyses about ccRCC in the Oncomine database. Expression of NR3C1 was examined in ccRCC and peritumoral tissues using RT-qPCR (n = 8) **D** and Western blot (n = 10) **E**. **F** Expression of NR3C1 was examined in ccRCC cells and normal renal tubular epithelial cells (HK2) using RT-qPCR. Four types of ccRCC cells are included: 786-O, ACHN, A498 and UMRC6. **G** Expression of NR3C1 was analyzed in 786-O, ACHN and HK2 cells using Western blot. Data presented as mean ± SD with three replicates. Ns, no significant difference; ***P < 0.001; ****P < 0.0001. ccRCC, clear cell Renal Cell Carcinoma; TCGA, The Cancer Genome Atlas; KIRC, kidney renal clear cell carcinoma; CPTAC, Clinical Proteomic Tumor Analysis Consortium; Oncomine, Oncomine tumor chip database; T, ccRCC tissues; P, peritumoral tissues

To investigate the expression level of NR3C1 in ccRCC, we first searched the The Cancer Genome Atlas (TCGA) and Clinical Proteomic Tumor Analysis Consortium (CPTAC) databases. The results showed that both mRNA and protein expression of NR3C1 were significantly higher in ccRCC tissues than in normal tissues (*P* < 0.001) (Fig. 1A, B). Consistent results were obtained through meta-analysis of the whole datasets in the Oncomine database (*P* < 0.0001) (Fig. 1C). Next, we detected the expression level of NR3C1 in fresh ccRCC tissues and peritumoral tissues, and the results confirmed that the mRNA and protein expression of NR3C1 were significantly higher in 60% of ccRCC tissues than in peritumoral tissues (Fig. 1D, E). We further tested the expression level of NR3C1 in four ccRCC cell lines (786-O, ACHN, A498, UMRC6) and normal renal tubular epithelial cells (HK2). The results showed that the mRNA and protein expression of NR3C1 in 786-O and ACHN cells were significantly higher than those in HK2 cells (Fig. 1F, G). The quantitative statistical results of Western blot in cells and tissues are presented in Additional file 1: Fig. S1A, B.

### Knockdown of NR3C1 inhibits the proliferation of ccRCC

To further investigate the effect of elevated NR3C1 expression on ccRCC, we constructed ccRCC stable transfection cell lines with NR3C1 knockdown and assessed the transfection efficiency using Western blot. Compared to the untransfected and control vector transfected groups, the expression of NR3C1 decreased by over 50% following transfection with the NR3C1 knockdown vector, with consistent results obtained from two cell lines (*P* < 0.01) (Additional file 1: Fig. S1C, D).

**Fig. 2:**
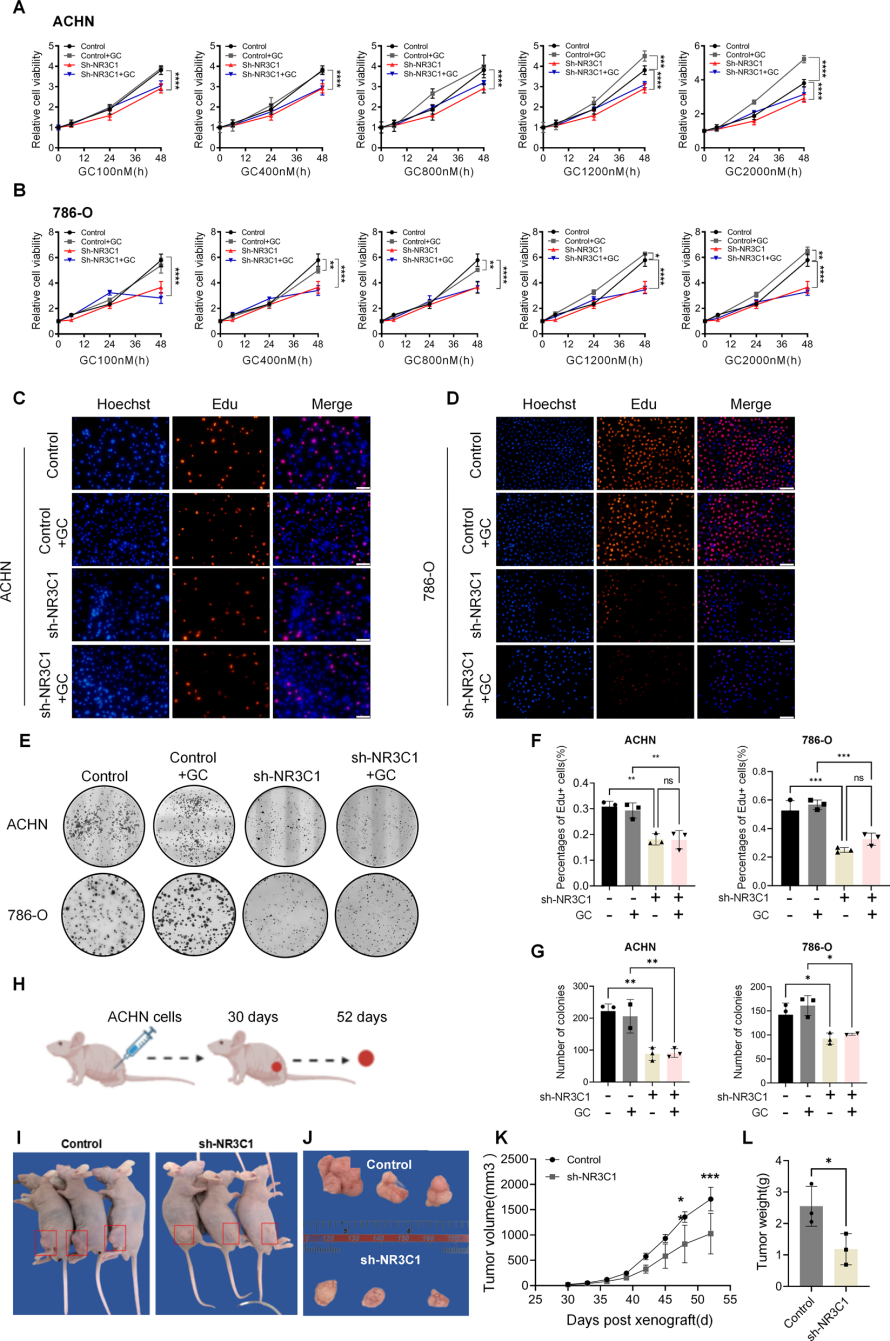
Knockdown of NR3C1 inhibits the proliferation of ccRCC. The effect of NR3C1 knockdown on the proliferation of ACHN** A** and 786-O cells **B** were assessed using CCK-8 assays. The concentrations of GC were: 100 nM, 400 nM, 800 nM, 1200 nM and 2000 nM. The effect of NR3C1 knockdown on the proliferation of ACHN **C** and 786-O cells **D** were detected using EdU assays. (GC = 1200 nM; scale bar  = 100 μm). **E** The effect of NR3C1 knockdown on the colony formation ability of ccRCC cells. (GC = 1200 nM). Bar graphs represent the results of EdU **F** and colony formation assays **G** for both ccRCC cells. **H** Schematic overview for **I**–**L**. **I**,** J** Images of subcutaneous tumor formation in nude mice at 7 weeks after injection of 2×10^5^ ACHN cells, with or without NR3C1 knockdown. Tumor volumes (**K**) and tumor weights (**L**) were measured for each group (n = 3). Data presented as mean ± SD with three replicates. Ns, no significant difference; *P< 0.05; **P<0.01; ***P< 0.001. GC, glucocorticoids

To characterize the effect of NR3C1 knockdown on the proliferation of ccRCC, we divided ACHN and 786-O cells into four groups based on whether glucocorticoids (GC) were added: control, control + GC, sh-NR3C1, and sh-NR3C1 + GC. We then performed CCK-8 assays with different concentrations of GC (100 nM, 400 nM, 800 nM, 1200 nM and 2000 nM). The results showed that the proliferation of the sh-NR3C1 group was significantly lower than that of the control group in both cell lines (*P* < 0.0001). Additionally, as the concentration of GC increased, the proliferation of cells in the control + GC group showed an increased proliferation rate than that of the control group. At a concentration of 1200 nM, the difference between the two groups was highly significant (*P* < 0.001), suggesting that this may be the basic GC concentration for activating the intracellular GR. However, there was no significant difference in cell proliferation between the sh-NR3C1 group and the sh-NR3C1 + GC group with increasing GC concentration (*P* > 0.05) (Fig. 2A, B). Based on these findings, a GC concentration of 1200 nM was selected for subsequent experiments. Furthermore, EdU assays confirmed that the cell proliferation of the sh-NR3C1 group was significantly lower than that of the control group (*P* < 0.01), and there was no significant difference between the sh-NR3C1 group and the sh-NR3C1 + GC group (*P* > 0.05) (Fig. 2C, D, F). Consistent results were also obtained in colony formation assays (Fig. 2E, G).

In addition, ACHN cells were divided into control and sh-NR3C1 groups and implanted subcutaneously in the left or right upper middle inguinal of nude mice to assess tumorigenesis (Fig. 2H, I). The results showed that knockdown of NR3C1 significantly slowed tumor growth in nude mice, with a final tumor weight of only about 40% of that in the control group (*P* < 0.05) (Fig. 2 J–L). These results demonstrate that knockdown of NR3C1 can inhibit the proliferation of ccRCC.

### Knockdown of NR3C1 inhibits the migration of ccRCC

**Fig. 3:**
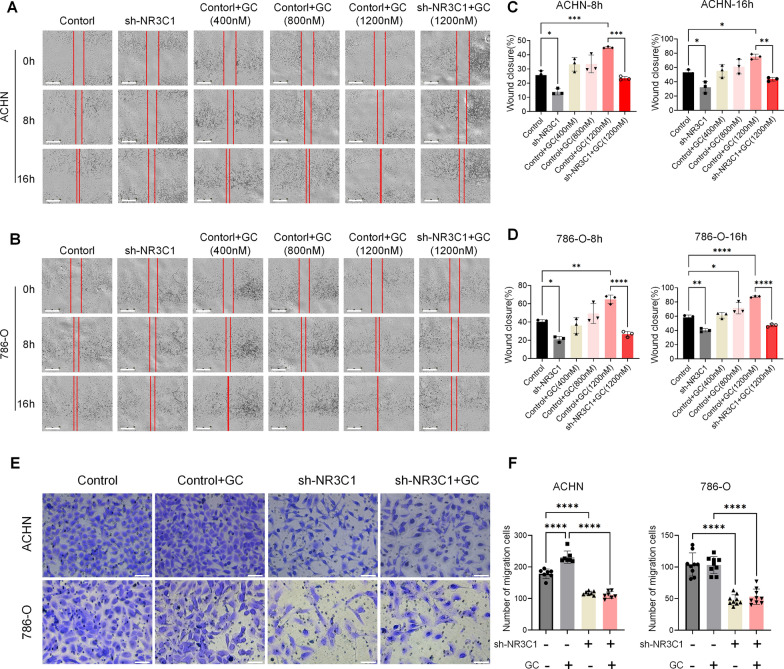
Knockdown of NR3C1 inhibits the migration of ccRCC. The effect of NR3C1 knockdown on the migration of ACHN (**A**) and 786-O cells (**B**) were assessed using wound healing assays. The concentrations of GC were: 400nM, 800 nM and 1200 nM. Images were acquired at 0, 8 and 16h. (Scale bar =700 μm). Bar graphs represent the changes in scratch healing area in ACHN (**C**) and 786-O cells (**D**) at 8 and 16h.** E** The effect of NR3C1 knockdown on the migration of ccRCC cells were assessed using Transwell assays. Images were acquired at 12h, and are representative of three independent experiments. (GC=1200 nM; scale bar =100 μm). **F** Bar graphs represent the results of Transwell assays in ACHN and 786-O cells. Data presented as mean  ±  SD with three replicates. *P <  0.05; **P <  0.01; ***P <  0.001; ****P <  0.0001. GC, glucocorticoids

At the same time, to investigate the effect of NR3C1 knockdown on ccRCC cell migration, we conducted wound healing assays with the following groups: control, sh-NR3C1, control + GC (400 nM), control + GC (800 nM), control + GC (1200 nM), and sh-NR3C1 + GC (1200 nM) group. The results showed that the cell migration rate in the sh-NR3C1 group was significantly lower than that in the control group at 8 and 16 h (*P* < 0.05). In the control group, the cell migration rate gradually increased with increasing GC concentration. At a concentration of 1200 nM, the difference between the two groups was statistically significant (8 h: *P* < 0.0001; 16 h: *P* < 0.01). However, once NR3C1 was knocked down, GC stimulation could not increase ccRCC cell migration (*P* > 0.05) (Fig. 3A–D). Transwell assays also confirmed that knockdown of NR3C1 inhibited ccRCC cell migration (*P* < 0.0001) (Fig. 3E, F).

### Lipidomic alterations in ccRCC cells after NR3C1 knockdown

**Fig. 4:**
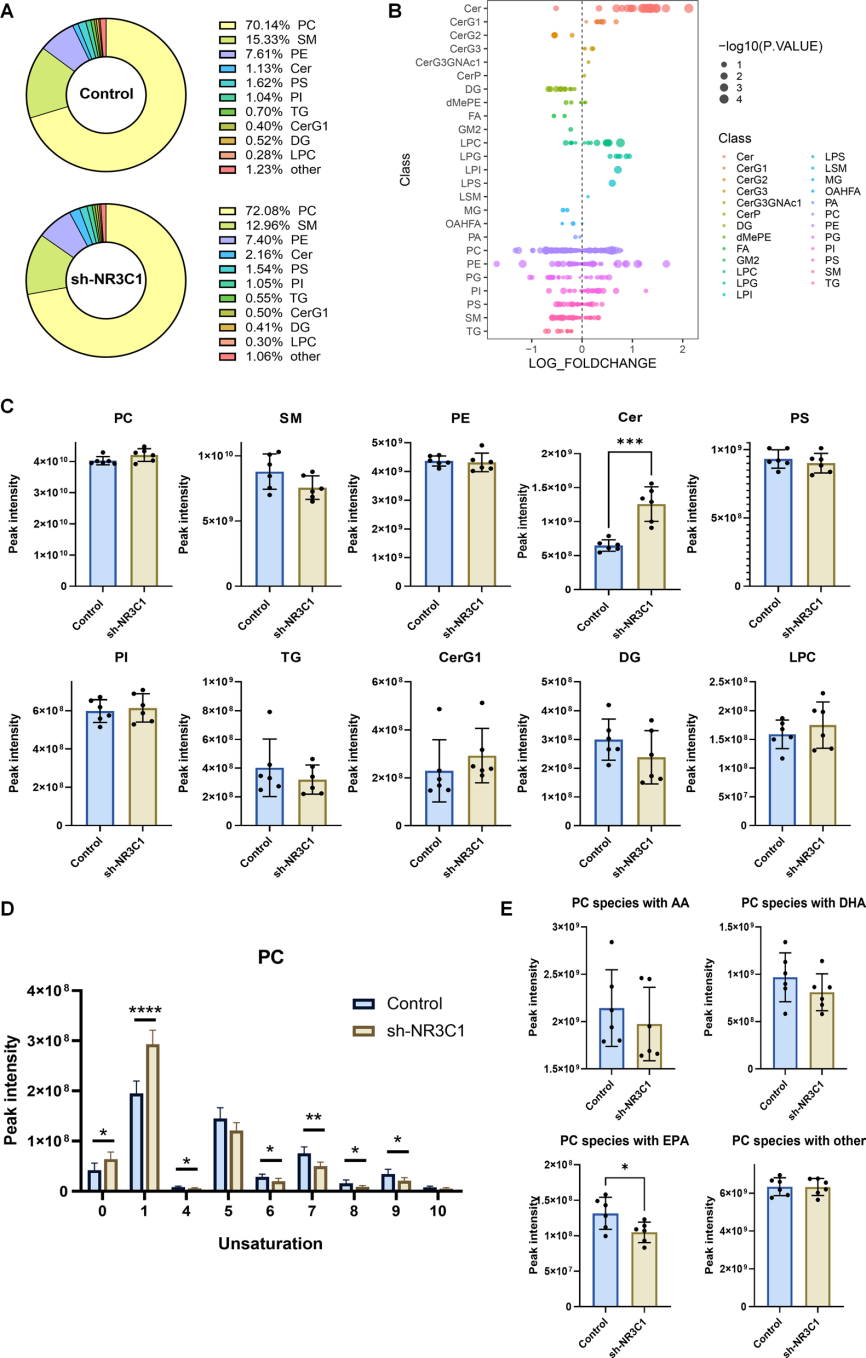
Lipidomic profiles in ccRCC cells after NR3C1 knockdown. **A** Metabolites donut charts of the control and sh-NR3C1 groups. Different colors represent different metabolite classification. **B** Bubble chart showed the differences in lipid metabolites levels between the control and sh-NR3C1 groups. Each point in the figure represents a different lipid, with the color of the point corresponding to different lipid subclasses and the size of the point indicating p after − log10 conversion. **C** Bar graphs illustrate the differences in expression of major lipid components between the two groups. **D** Chain unsaturation distribution of PC in the control and sh-NR3C1 groups. The x-axis represents the unsaturation of the carbon chain, while the y-axis represents peak intensity. The numbers on the x-axis indicate the number of unsaturated fatty acids present in the carbon chain. **E** Bar graphs illustrate the differences in expression of classical polyunsaturated fatty acids profiles between the control and sh-NR3C1 groups. Data presented as mean  ±  SD with three replicates. *P < 0.05; **P < 0.01; ***P < 0.001; ****P < 0.0001. PC, phosphatidylcholine; SM, sphingomyelin; PE, phosphatidylethanolamine; Cer, ceramides; PS, phosphatidylserine; PI, phosphatidylinositol; TG, triglyceride; CerG1, monogylcosylceramide; DG, diglyceride; LPC, lyso-phosphatidylcho line; AA, arachidonic acid; DHA, docosahexaenoic acid; EPA, eicosapentaenoic acid

As the sole gene responsible for encoding GR, NR3C1 is closely related to glycan and lipid metabolism. Therefore, we conducted lipidomics sequencing with ACHN cells to investigate changes in lipid metabolism after NR3C1 knockdown in ccRCC cells. We identified a total of 541 lipid metabolites through lipidomics sequencing. The circular diagram of metabolite classification illustrated that the distribution of lipid metabolites was comparable between the sh-NR3C1 and control group. Photoshatidylcholine (PC) accounted for the highest proportion in both groups, with more than 70% (Fig. 4A). Further analysis of differentially expressed lipid metabolites revealed that there were 38 up-regulated and 24 down-regulated lipid metabolites after NR3C1 knockdown, of which Ceramide (Cer) was most significantly up-regulated (*P* < 0.001) (Fig. 4B, C).

In addition to the change in lipid metabolite composition, alterations in chain unsaturation of lipid molecules are also closely related to malignancies. In the unsaturation analysis of PC, we found that the content of polyunsaturated fatty acids (PUFAs) on the second chain of PC was significantly decreased after NR3C1 knockdown, indicating a decrease in the overall unsaturation of PC (Fig. 4D). Further analysis of the most common PUFAs showed that the content of arachidonic acid (AA), docosahexaenoic acid (DHA), and eicosapentaenoic acid (EPA) were decreased in the sh-NR3C1 group, and the difference in EPA content between the two groups was statistically significant (*P* < 0.05) (Fig. 4E).

It has been reported that excessive intracellular accumulation of Cer can induce mitophagy and lead to cell death in breast cancer and Parkinson's disease [18, 19]. Additionally, decreased intracellular lipid unsaturation can activate ER stress and induce the unfolded protein response [20]. Therefore, we speculate that ER stress and mitophagy may occur in ccRCC cells after NR3C1 knockdown.

### The mRNA expression profiles in ccRCC cells after NR3C1 knockdown

**Fig. 5:**
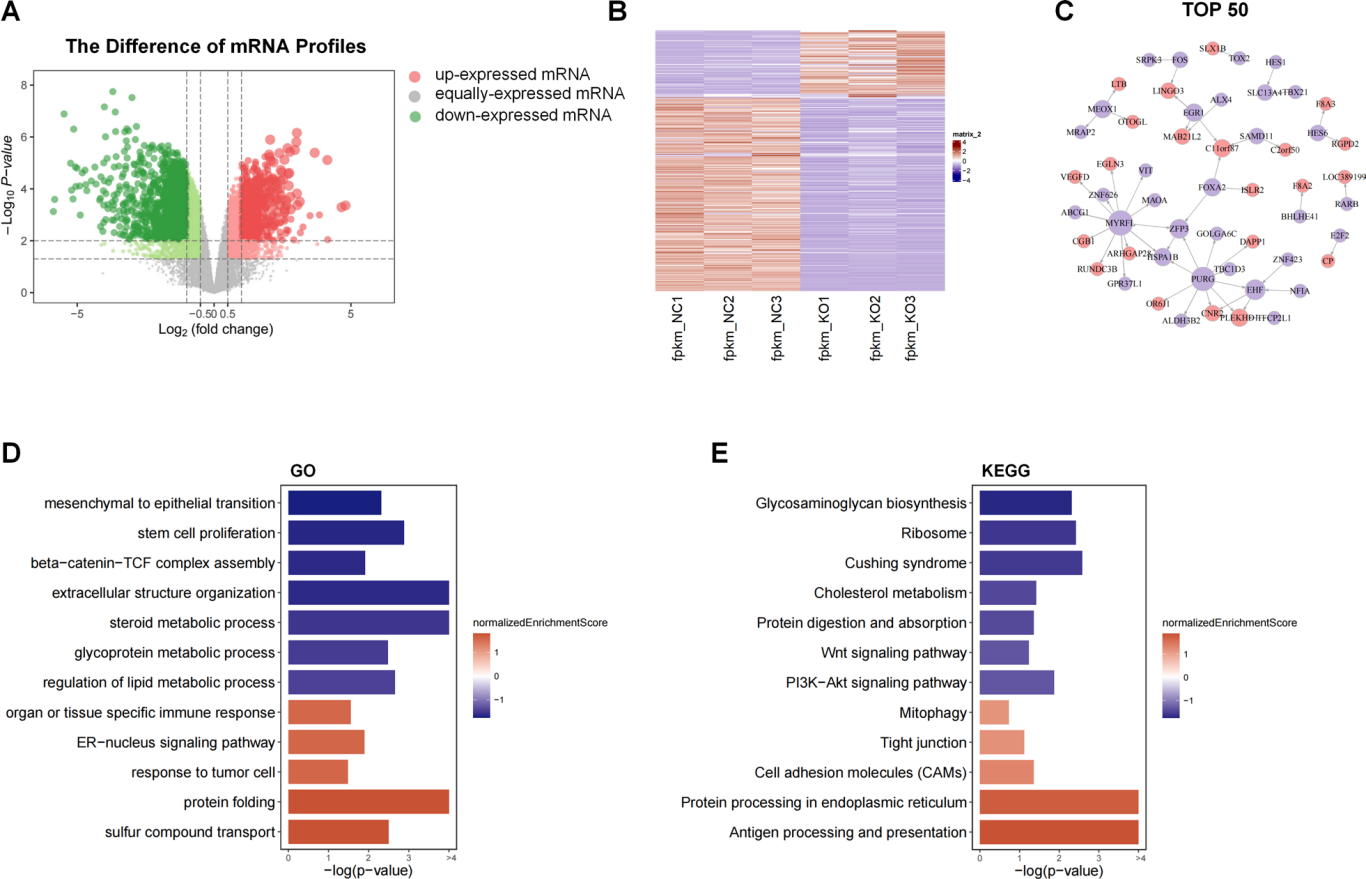
The mRNA expression profiles in ccRCC cells after NR3C1 knockdown. **A** Volcano map of diferentially expressed genes (DEGs) between the control and sh-NR3C1 groups. Red represents up-regulated DEGs, blue represents down-regulated DEGs, and gray represents non-DEGs. **B** The heatmap diagram illustrates the expression levels of DEGs in each group, with a gradient from blue to red indicating down-regulation to up-regulation of the genes. **C** Transcription factor regulatory network of DEGs between two groups. GO (**D**) and KEGG (**E**) pathway analysis of DEGs. Red represents up-regulated enriched pathways, blue represents down-regulated enriched pathways. DEGs, differentially expressed genes; GO, Gene Ontology; KEGG, Kyoto Encyclopedia of Genes and Genomes

To investigate whether these changes occurred in ccRCC cells after NR3C1 knockdown, we conducted transcriptomic sequencing of ACHN cells. A total of 16,489 genes were detected in this study, and the volcano plot showed no significant difference in the number of up-regulated and down-regulated DEGs in ccRCC cells after NR3C1 knockdown (Fig. 5A). The clustering heatmap indicated that the DEGs clustered by different samples in the two groups were consistent, and the samples clustered by different genes are also basically the same (Fig. 5B). Furthermore, as activated NR3C1 can act as a transcription factor or co-regulator, we constructed a mutual regulatory network of transcription factors based on the sequencing data. We found that genes such as myelin regulatory factor like (MYRFL), early growth response 1 (EGR1), zinc finger protein3 (ZFP3), and mesenchyme homeobox 1 (MEOX1) played key roles in the mutual regulation of transcription factors after NR3C1 knockdown (Fig. 5C).

Next, we conducted functional enrichment analysis of DEGs based on GO and KEGG databases. The results showed that the regulation of lipid metabolism process and cholesterol metabolism were down-regulated after NR3C1 knockdown, indicating a possible disorder of lipid metabolism. Conversely, protein folding, ER-nucleus signaling pathway, and sulfur compound transport and protein processing in the ER were up-regulated, suggesting the presence of ER stress. Moreover, up-regulation of mitophagy was also enriched, indicating changes in mitochondrial function (Fig. 5D, E). Taken together, these results suggest that knockdown of NR3C1 may lead to disturbed lipid metabolism in ccRCC cells, activating ER stress and mitophagy.

### Knockdown of NR3C1 activates ER stress and mitophagy in ccRCC

**Fig. 6:**
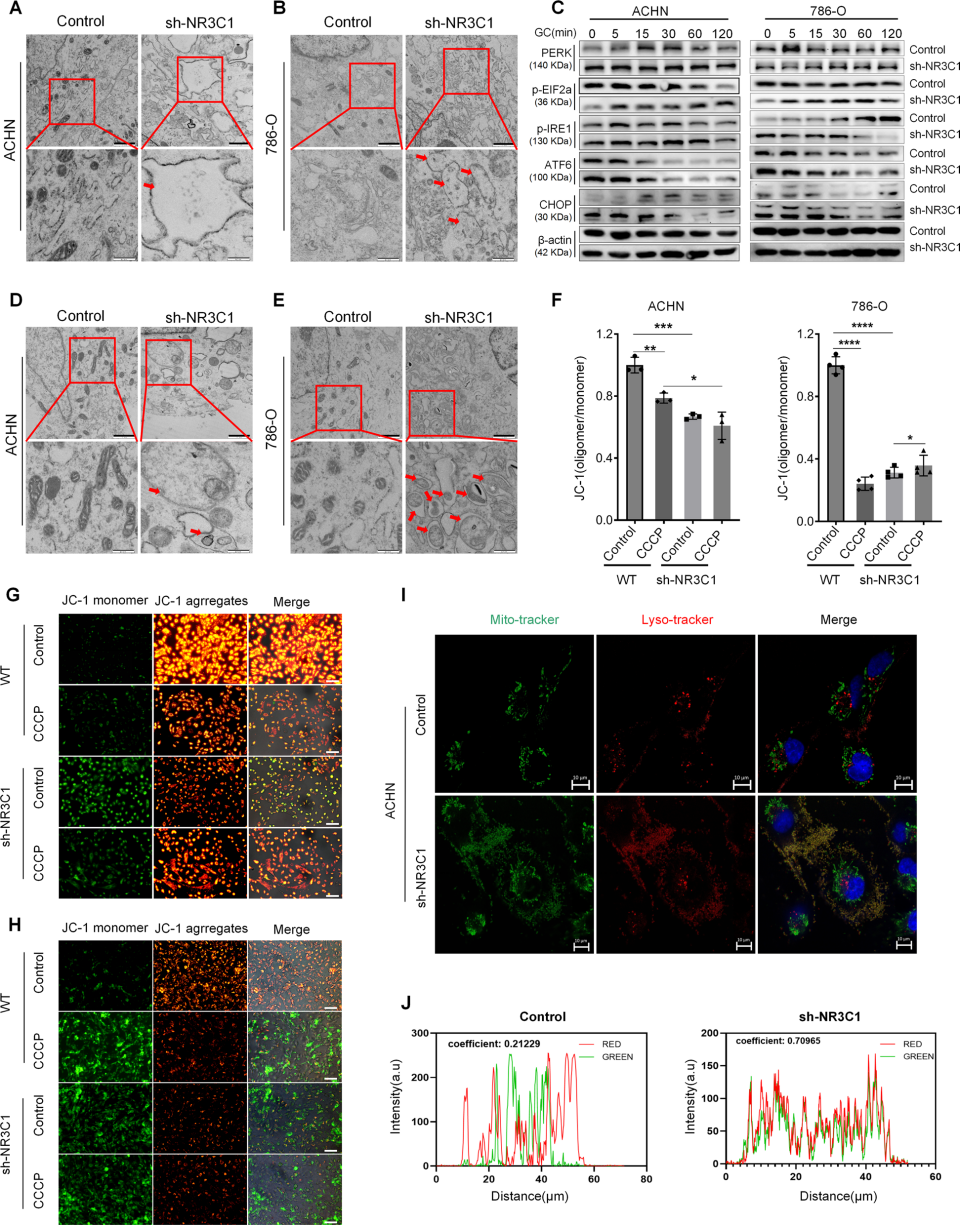
Knockdown of NR3C1 activates ER stress and mitophagy in ccRCC. TEM of ER stress in the control and sh-NR3C1 groups of ACHN (**A**) and 786-O (**B**) cells. Shown are representative images, with arrows indicating ER stress. (Scale bar =1.0 μm; enlarged scale bar =0.4 μm). **C** Expression levels of ER stress pathway proteins in the two groups of ACHN and 786-O cells at different times of GC treatment using Western blot. (GC=1200 nM). TEM of mitophagy in the control and sh-NR3C1 groups of ACHN (**D**) and 786-O (**E**) cells. Shown are representative images, with arrows indicating mitochondrial autophagosomes. (Scale bar =1.0 μm; enlarged scale bar =0.4 μm). **F** Bar graphs represent the statistical results of mitochondrial membrane potential detection. Representative fluorescence images of mitochondrial membrane potential in the control and sh-NR3C1 groups of ACHN (**G**) and 786-O (**H**) cells were stained by the JC-1 probe, with CCCP used as a positive control. (Scale bar =100 μm). **I** Colocalization of lysosomes and mitochondria in ACHN cells after NR3C1 knockdown was determined by confocal microscopy. Mitochondria and lysosomes were labeled with mitotracker and lysotracker, respectively. (Scale bar =10 μm).** J** The overlap coefficient of immunofluorescence co-localization. Data presented as mean ± SD with three replicates. *P < 0.05; **P < 0.01; ***P < 0.001; ****P < 0.0001. TEM, transmission electron microscopy; GC, glucocorticoids; CCCP, carbonylcyanide-m-chlorophenylhydrazone; Mito-tracker, green fluorescent probe of the mitochondria; Lyso-tracker, red fluorescent probe of lysosome

To validate the results of transcriptomics and lipidomics sequencing, we first used TEM to observe ACHN and 786-O cells and found significant swelling and expansion in the sh-NR3C1 group, with the normal folded structures in the ER disappearing. This finding proved that knockdown of NR3C1 activated ER stress in ccRCC cells (Fig. 6A, B). We then stimulated ccRCC cells with 1200 nM GC for 0–120 min to further explore the signaling pathway alterations after ER stress. Western blot was used to detect the protein expression of each pathway of ER stress. The results showed that ATF6 and CHOP expression were significantly higher in the sh-NR3C1 group than in the control group before 30 min (*P* < 0.01). However, their expression decreased significantly from 30 min, indicating that ATF6 and CHOP may have been degraded after 30 min (Fig. 6C). The statistical results are shown in Additional file 1: Fig. S2A, B. These findings demonstrate that the knockdown of NR3C1 activated the ATF6-CHOP pathway.

Next, we observed mitochondrial morphology in ccRCC cells using TEM and found a large number of mitochondrial autophagosomes after knockdown of NR3C1 (Fig. 6D, E). After detecting changes in mitochondrial membrane potential, we found that the fluorescence of ccRCC cells in the sh-NR3C1 group changed from red to green, with a trend similar to that of the positive control group of carbonylcyanide-m- chlorophenylhydrazone (CCCP), indicating that knockdown of NR3C1 induced mitochondrial membrane potential damage in ccRCC cells (*P* < 0.001) (Fig. 6F–H). Finally, we performed immunofluorescence co-localization experiments with mitotracker and lysotracker to visualize the fusion process of mitochondrial autophagosome and lysosome. The results showed that the green fluorescence of mitotracker overlapped with the red fluorescence of lysotracker in the sh-NR3C1 group, with an overlap coefficient of 0.70965, which was significantly higher than that of the control group (Fig. 6I, J). Together, these findings confirmed that knockdown of NR3C1 could induce mitophagy in ccRCC cells.

### Knockdown of NR3C1 activates mitophagy in ccRCC through ATF6-PINK1/BNIP3 pathway

**Fig. 7:**
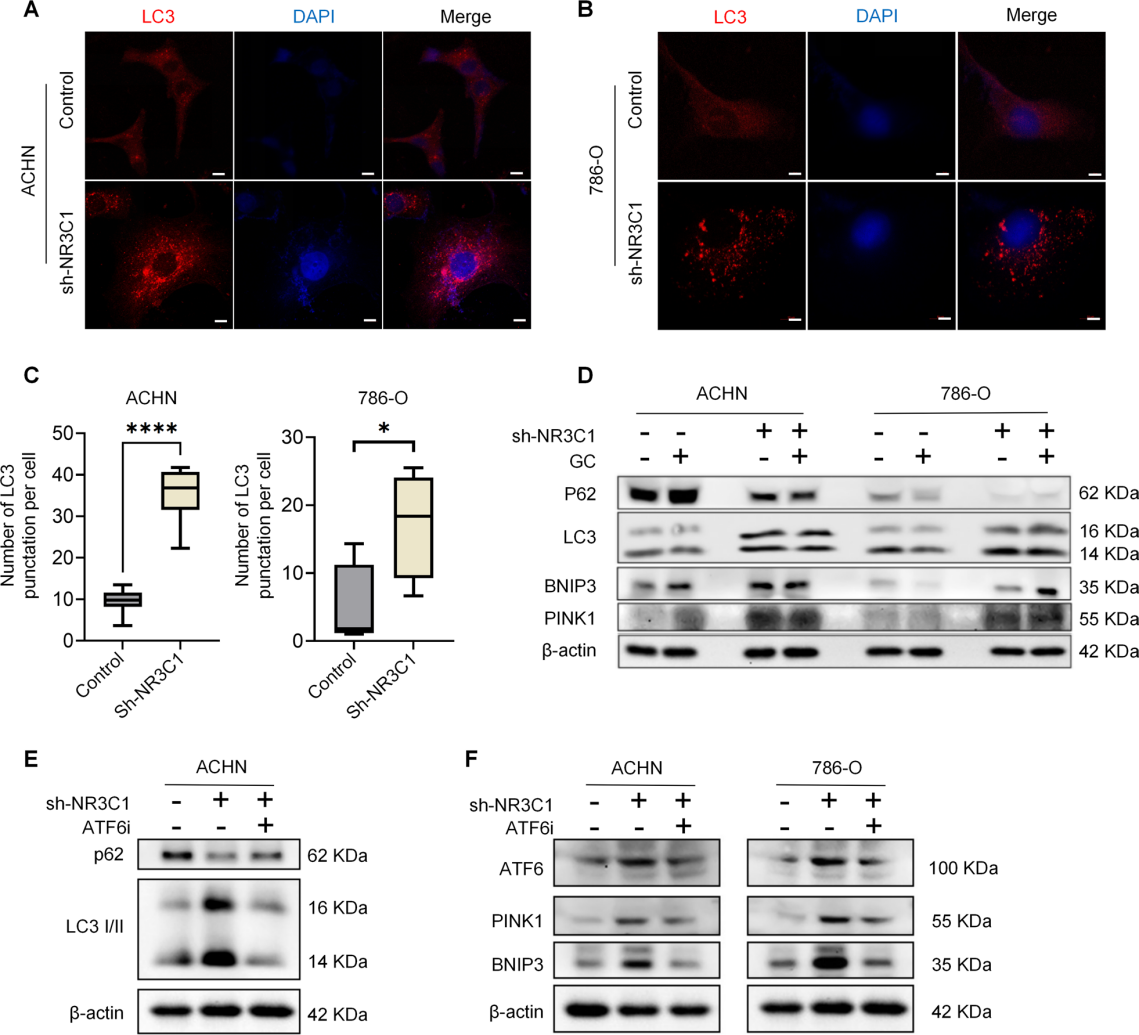
Knockdown of NR3C1 activates mitophagy in ccRCC through ATF6-PINK1/BNIP3 pathway. Expression of LC3 in the control and sh-NR3C1 groups of ACHN (**A**) and 786-O (**B**) cells by immunofuorescence. (Scale bar =10 μm). The statistical results are shown in **C**. **D** Expression levels of P62, LC3, BNIP3 and PINK1 in four different groups of ACHN and 786-O cells were measured using Western blot. The four groups are: control, control+GC, sh-NR3C1 and sh-NR3C1+GC group. (GC = 1200 nM). **E** Expression levels of P62 and LC3 in three different groups of ACHN cells were measured using Western blot. The three groups are: control, sh-NR3C1 and sh-NR3C1+ATF6i (Ceapin-A7) group. **F** Expression levels of ATF6, PINK1 and BNIP3 in three different groups of ACHN and 786-O cells were measured using Western blot. Three groups were: control, sh-NR3C1, sh-NR3C1+ATF6i. Data presented as mean  ±  SD with three replicates. *P < 0.05; ***P <  0.001. LC3, microtubule associated protein light chain 3; GC, glucocorticoids; ATF6i, Ceapin-A7 as ATF6 inhibitor

Microtubule associated protein light chain 3 (LC3) is commonly used as a marker to evaluate the occurrence of autophagy. Through cellular immunofluorescence staining, we observed a significantly higher number of LC3 fluorescent spots in the sh-NR3C1 group compared to the control group (786-O group: *P* < 0.05; ACHN group: *P* < 0.0001) (Fig. 7A–C). Furthermore, we detected the expression of proteins involved in mitophagy pathways by Western blot. Specifically, cells in the GC group were treated with 1200 nM GC for 24 h. The results indicated that the expression of LC3, BNIP3, and PINK1 significantly increased in the sh-NR3C1 group, while the expression of P62 significantly decreased, and the differences between the two groups were statistically significant (*P* < 0.05) (Fig. 7D). The statistical results are shown in Additional file 1: Fig. S2C, D. These results suggest that knockdown of NR3C1 induces mitophagy in ccRCC cells by activating BNIP3 and PINK1.

To clarify the relationship between ER stress and mitophagy, we used 20 μM Ceapin-A7, a specific inhibitor of ATF6 activation that functions by competitively binding to ATF6 and preventing its translocation to the Golgi apparatus [21], to stimulate cells in the sh-NR3C1 group for 5 h. Western blot was used to detect changes in mitophagy pathways in each group. The results showed that after the addition of Ceapin-A7, the previously increased LC3 in the sh-NR3C1 group of ACHN cells significantly decreased, while the previously decreased P62 increased significantly, and the difference between the two groups was statistically significant (*P* < 0.05) (Fig. 7E). Additionally, adding Ceapin-A7 could also reverse the original expression trend of PINK1 and BNIP3 in the sh-NR3C1 group (*P* < 0.05), and the results were consistent in both groups (Fig. 7F). The statistical results are shown in Additional file 1: Fig. S3. These findings confirm that ATF6 plays an important role in the regulation of PINK1 and BNIP3 in ccRCC cells with NR3C1 knockdown, and the primary cause of ER stress and mitophagy in ccRCC is the activation of ATF6.

### Knockdown of NR3C1 inhibits the proliferation and migration of ccRCC by activating ATF6

**Fig. 8:**
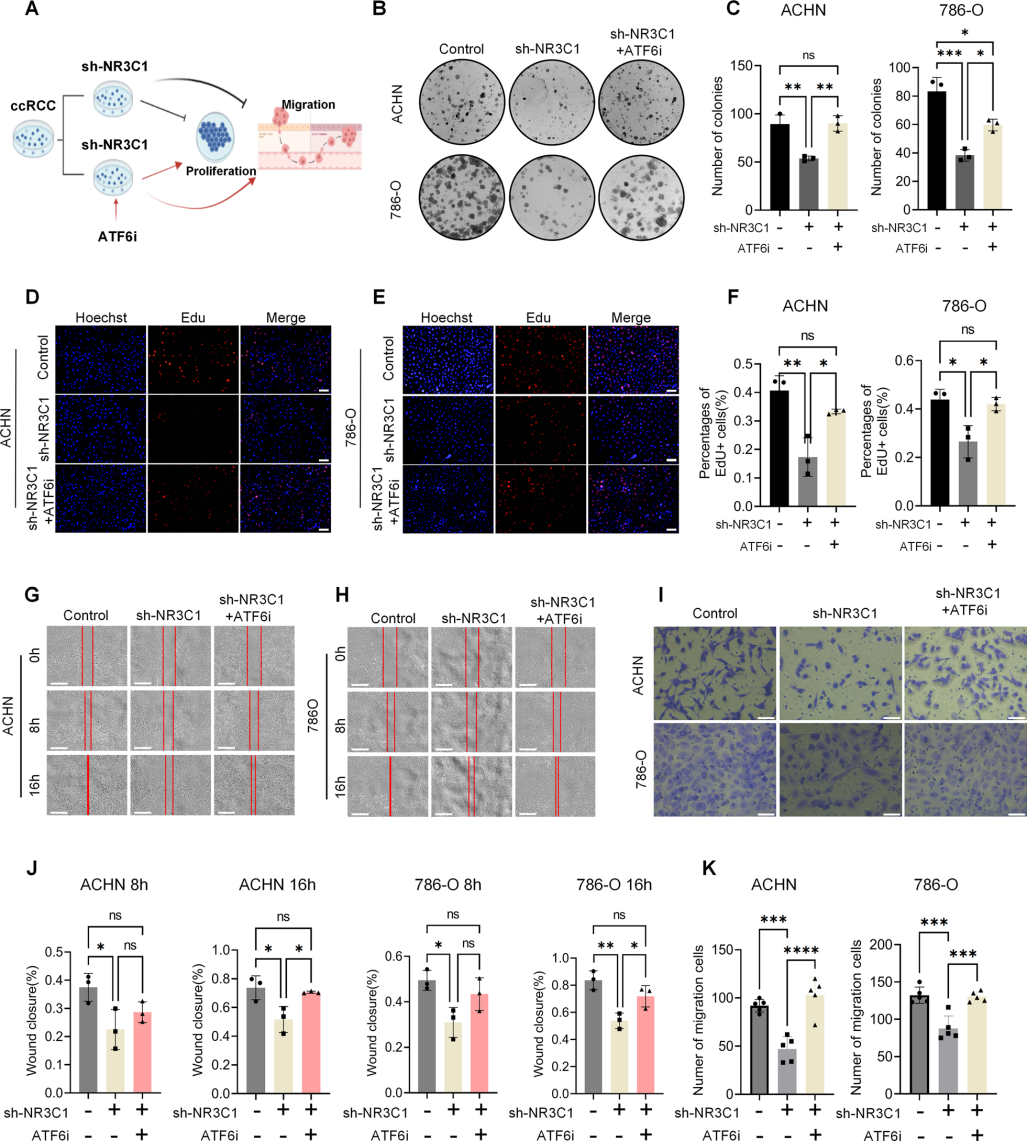
Knockdown of NR3C1 inhibits the proliferation and migration of ccRCC by activating ATF6. **A** Schematic overview for **B**–**K**. **B** Inhibition of ATF6 rescued the NR3C1 knockdown-mediated reduced colony formation ability of ACHN and 786-O cells, as assessed using clone formation assays. (Scale bar =100 μm). **C** Bar graphs represent the colony formation results in ACHN and 786-O cells. Inhibition of ATF6 rescued the NR3C1 knockdown-mediated altered proliferative capacity of ACHN (**D**) and 786-O (**E**) cells, as assessed using EdU assays. (Scale bar =100 μm). **F** Bar graphs represent the EdU results in ACHN and 786-O cells. Inhibition of ATF6 rescued the NR3C1 knockdown-mediated altered migration ability of ACHN (**G**) and 786-O (**H**) cells, as assessed using wound healing assays. Images were acquired at 0, 8 and 16h. (Scale bar =700 μm). **I** Inhibition of ATF6 rescued the NR3C1 knockdown-mediated reduced migration ability of ACHN and 786-O cells, as assessed using Transwell assays. Images were acquired at 12h. (Scale bar =100 μm). **J** Bar graphs show the scratch healing area in ACHN and 786-O cells at 8 and 16h. **K** Bar graphs represent the results of Transwell assays in ACHN and 786-O cells. The cells in the rescue group were pretreated with 20 µM ATF6i for 24h. Data presented as mean  ±  SD with three replicates. Ns, no significant difference; *P <  0.05; **P <  0.01; ***P <  0.001; ****P <  0.0001. ATF6i, Ceapin-A7 as ATF6 inhibitor

Finally, we conducted a rescue experiment targeting ATF6 to explore its effect on the proliferation and migration of ccRCC. We divided ccRCC cells into three groups: control, sh-NR3C1, and sh-NR3C1 + ATF6i (Ceapin-A7) group. The cells in the sh-NR3C1 + ATF6i group were pre-treated with 20 µM Ceapin-A7 for 24 h. Figure 8A provides a schematic overview of this experimental design. As shown by the clone formation assay (Fig. 8B, C) and EdU assay (Fig. 8D-F), the addition of Ceapin-A7 significantly reversed the previously reduced proliferation of ccRCC cells in the sh-NR3C1 group (*P* < 0.05). In the wound healing assay (Fig. 8G, H, J) and Transwell assay (Fig. 8I, K), adding Ceapin-A7 also improved the migration of ccRCC cells in the sh-NR3C1 group (*P* < 0.05). Taken together, these findings suggest that knockdown of NR3C1 may inhibit the proliferation and migration of ccRCC cells by activating ATF6.

## Discussion

NR3C1 is a crucial molecule in the common steroid hormone-receptor signaling pathway, and its role in malignancies has received widespread attention recently. It has been demonstrated that NR3C1 plays a vital role in the development of various malignancies, such as triple-negative breast cancer, ovarian cancer, and urothelial cancer [11–14]. Although ccRCC is considered to be a malignancy closely related to hormones, there is still limited research on the correlation between NR3C1 and ccRCC. It is currently unclear whether NR3C1 has a carcinogenic role in ccRCC and by what mechanism it affects ccRCC. Our study confirmed the elevated expression of NR3C1 in ccRCC and found that knockdown of NR3C1 inhibited the proliferation and migration of ccRCC. We also performed transcriptomics sequencing, lipomics sequencing, and molecular biology experiments, which further confirmed that the intrinsic mechanism affecting ccRCC after knockdown of NR3C1 is activation of ER stress-mitophagy. To the best of our knowledge, this is the first research that establishes a link between NR3C1 and ER stress-mitophagy in ccRCC.

In this study, we first validated the increased expression of NR3C1 in ccRCC, which is consistent with the reports of Arai [22] and Czarnecka [7]. By detecting different ccRCC cell lines, we confirmed that NR3C1 expression was most significantly elevated in 786-O and ACHN cells, which are considered classic cell lines of ccRCC due to their positive expression of PBRM1 [23, 24], providing a basis for selecting these two cell lines for subsequent experiments. It is worth noting that Yakirevich [25] reported that the elevated expression of NR3C1 was present in most ccRCC tissues and was rare in other pathological types. This implies the pathological type specificity and possibly unique role of NR3C1 in ccRCC.

It has been reported that ccRCC is closely related to lipid metabolism disorders [26], and NR3C1, a key molecule in the GC-GR signaling pathway, plays an important role in regulating carbohydrate and lipid metabolism [27–29]. Through differential expression analysis of lipid metabolites, we found that knockdown of NR3C1 resulted in a significant up-regulation of Cer, a vital member of the neurosphingolipids with potent activity to regulate intracellular proteins [30]. A large number of studies have confirmed that the excessive accumulation of Cer can lead to the death of various cancer cells by inducing mitophagy [18, 19].

In addition to the above lipid composition alterations, we also found that the overall unsaturation of PC, which accounts for the highest proportion of lipid metabolites, decreased significantly after knocking down NR3C1. It is known that the higher the unsaturation, the greater the membrane fluidity, which is also proportional to cell migration [31, 32]. In this study, the decreased unsaturation of PC may lead to decreased membrane fluidity, thus reducing cell migration, which may be the main reason for the inhibition of cell migration after NR3C1 knockdown. Moreover, it has been reported that decreased lipid unsaturation can activate ER stress and subsequently induce the unfolded protein response [20]. While reduction of unsaturated fatty acids can lead to continuous ER stress in tumor cells, which can be effectively alleviated by appropriate supplementation of exogenous unsaturated fatty acids [33]. The above studies suggest that decreased lipid unsaturation in cells is an independent factor in inducing ER stress. Our transcriptomics sequencing results also verified this hypothesis: protein folding and ER-nucleus signaling pathway were positive enriched. Our results confirmed that the occurrence of ER stress was due to the activation of ATF6-CHOP pathway.

Furthermore, we enriched the up-regulation of mitophagy pathway by KEGG analysis, and observed a increased number of mitophagosomes, severely impaired mitochondrial membrane potential, and the fusion of mitophagosomes and lysosomes in the sh-NR3C1 group. Western blot further confirmed that knockdown of NR3C1 activated PINK1 and BNIP3 pathways, which are the most classic pathways of mitophagy. The simultaneous activation of both pathways reflects the severity of mitophagy. Numerous studies have demonstrated that excessive activation of mitophagy can lead to the death of various tumor cells [34–36]. PINK1 can inhibit the growth of several tumors by activating mitophagy [37, 38]. Meanwhile, BNIP3, activated by Cer, can induce mitophagy and lead to the death of malignant glioma cells [39]. Similar researches have also been reported in esophageal cancer and lung cancer [40, 41].

Recently, several studies have demonstrated the ability of ER stress to activate mitophagy [42–44]. In this study, we conducted rescue experiments and found that in ccRCC cells with knockdown of NR3C1, ATF6 is a direct regulator for the expression of PINK1 and BNIP3, and is also a key protein in mitophagy induced by ER stress. Activating ATF6 has been reported to induce apoptosis by inducing mitochondrial dysfunction [45, 46], which partly supports our finding. Finally, we confirmed through cell functional rescue assays that the reduced proliferation and migration of ccRCC cells caused by knocking down NR3C1 were effectively reversed by inhibiting ATF6.

In summary, this study demonstrated for the first time that knockdown of NR3C1 activates ER stress-mitophagy through the ATF6-PINK1/BNIP3 pathway, leading to decreased proliferation and migration of ccRCC. However, our study has several limitations. Firstly, although it has been reported that elevated NR3C1 expression is associated with poor prognosis in other malignancies, the relationship between NR3C1 expression and prognosis in ccRCC is still unclear. The results of survival analysis in different databases are inconsistent, and only one patient included in this study has died, so the survival analysis cannot be conducted at present. This should be further verified by increasing the number of patients or extending the follow-up period in future studies. Secondly, this study confirmed through lipidomic sequencing that there is lipid metabolism disorder in ccRCC cells after knocking down NR3C1. Although it has been reported to be closely related to ER stress, the correlation between the two has not been verified in this study. Finally, this study only proposed the theoretical possibility for treating ccRCC by targeting ER stress-mitophagy, but how to conduct drug researches on NR3C1, ATF6, PINK1 and BNIP3 are the focus of future research.

## Conclusions

In summary, this study confirms the elevated expression of NR3C1 in ccRCC. Knockdown of NR3C1 activates ER stress and induces mitophagy through the ATF6-PINK1/BNIP3 pathway, resulting in reduced proliferation and migration of ccRCC.

### Supplementary Information

**Additional file 1: Figure S1.** Expression levels of NR3C1 in ccRCC. **Figure S2.** Expression levels of ER stress and mitophagy signaling pathway proteins. **Figure S3.** Knockdown of NR3C1 activates mitophagy in ccRCC through ATF6-PINK1/BNIP3 pathway. **Figure S4.** Effect of NR3C1 knockdown on proliferation and migration of HK2 cells. **Figure S5.** The effect of NR3C1 knockdown on the oxygen consumption rate (OCR) in ccRCC cells. **Figure S6.** Differences in NR3C1 expression between ccRCC cells and HK2 cells.

**Additional file 2: Table S1.** Clinicopathological characteristics of ccRCC patients.

## Data Availability

All other relevant data supporting the key findings of this study are available within the article and its supplementary information files or from the corresponding author upon reasonable request.
