# Supplementary material for: Knockdown of NR3C1 inhibits the proliferation and migration of clear cell renal cell carcinoma through activating endoplasmic reticulum stress–mitophagy
Source: J Transl Med. 2023 Oct 8;21:701. doi: 10.1186/s12967-023-04560-2 (PMC10560440; doi:10.1186/s12967-023-04560-2)
Supplement: Supplementary file 1 — Additional file 1: Figure S1. Expression levels of NR3C1 in ccRCC. Figure S2. Expression levels of ER stress and mitophagy signaling pathway proteins. Figure S3. Knockdown of NR3C1 activates mitophagy in ccRCC through ATF6-PINK1/BNIP3 pathway. Figure S4. Effect of NR3C1 knockdown on proliferation and migration of HK2 cells. Figure S5. The effect of NR3C1 knockdown on the oxygen consumption rate (OCR) in ccRCC cells. Figure S6. Differences in NR3C1 expression between ccRCC cells and HK2 cells. [file 12967_2023_4560_MOESM1_ESM.pdf]

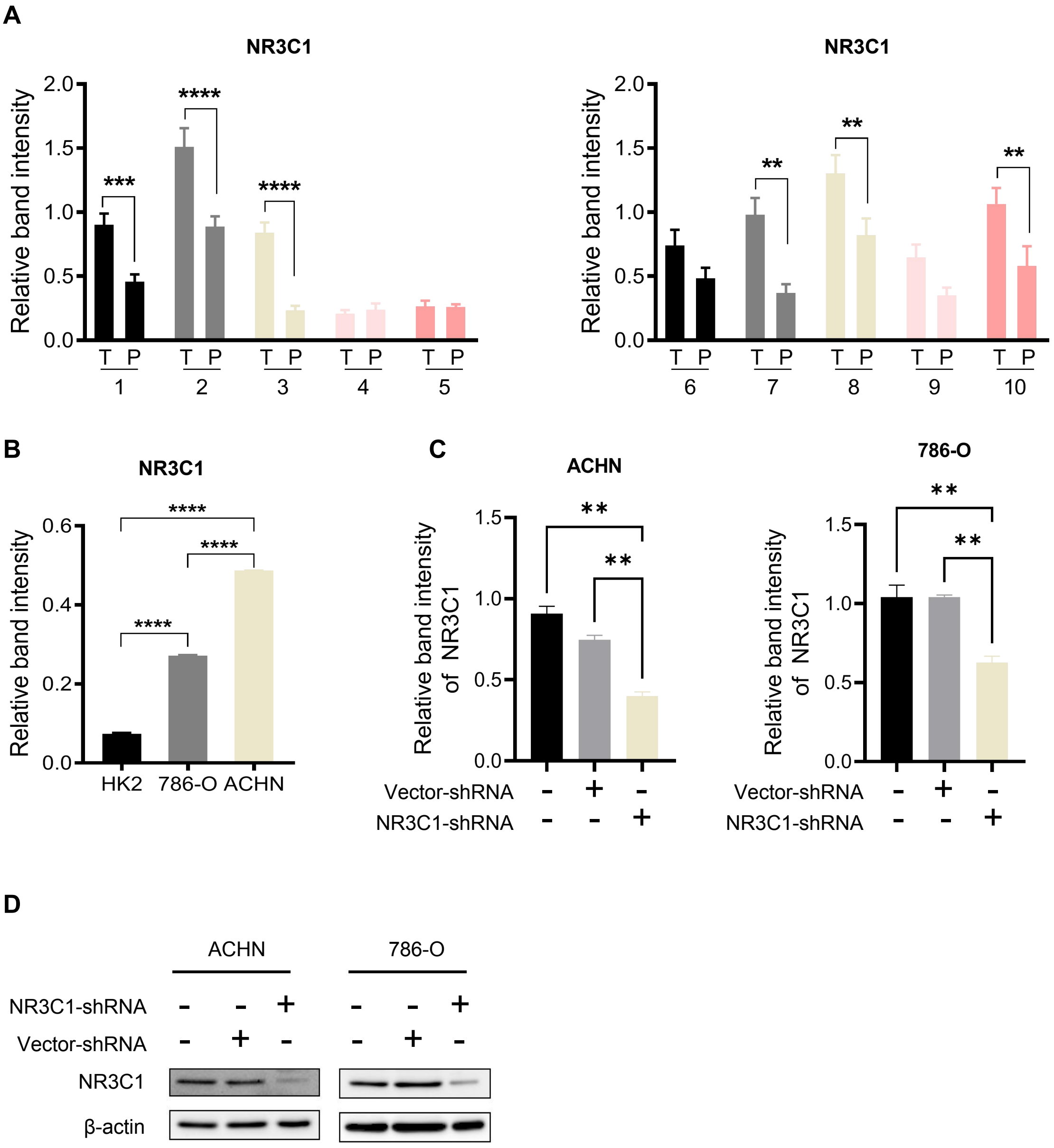

Supplement figure 1 Expression levels of NR3C1 in ccRCC.

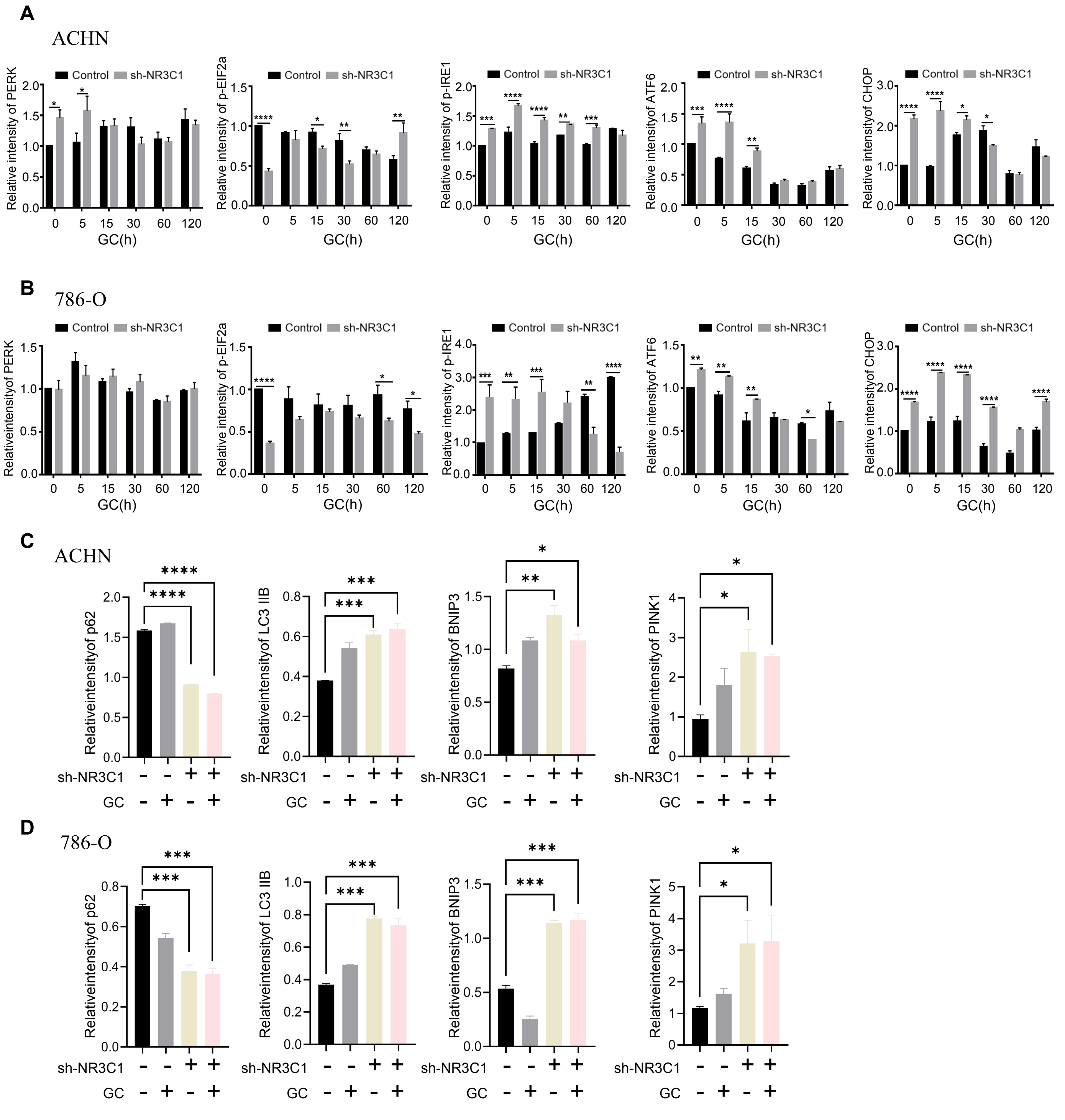

Supplement figure 2 Expression levels of ER stress and mitophagy signaling pathway proteins.

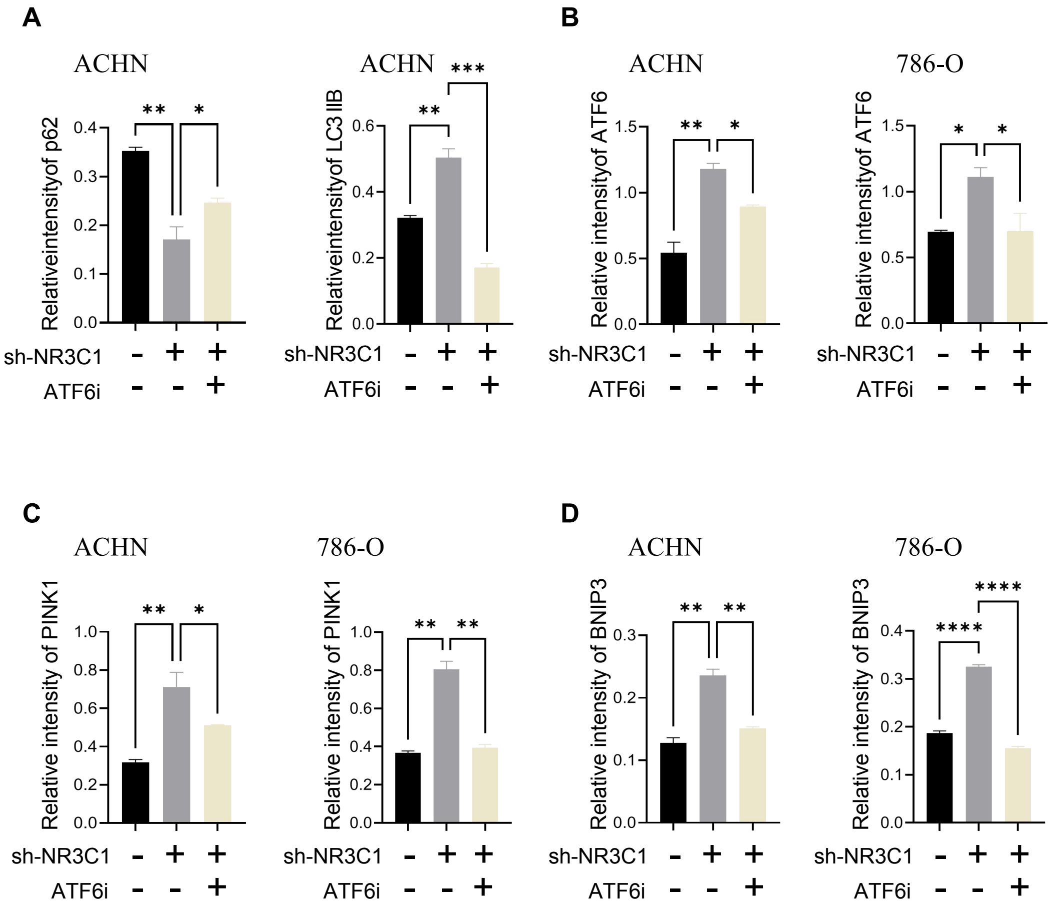

Supplement figure 3 Knockdown of NR3C1 activates mitophagy in ccRCC through ATF6-PINK1/BNIP3 pathway.

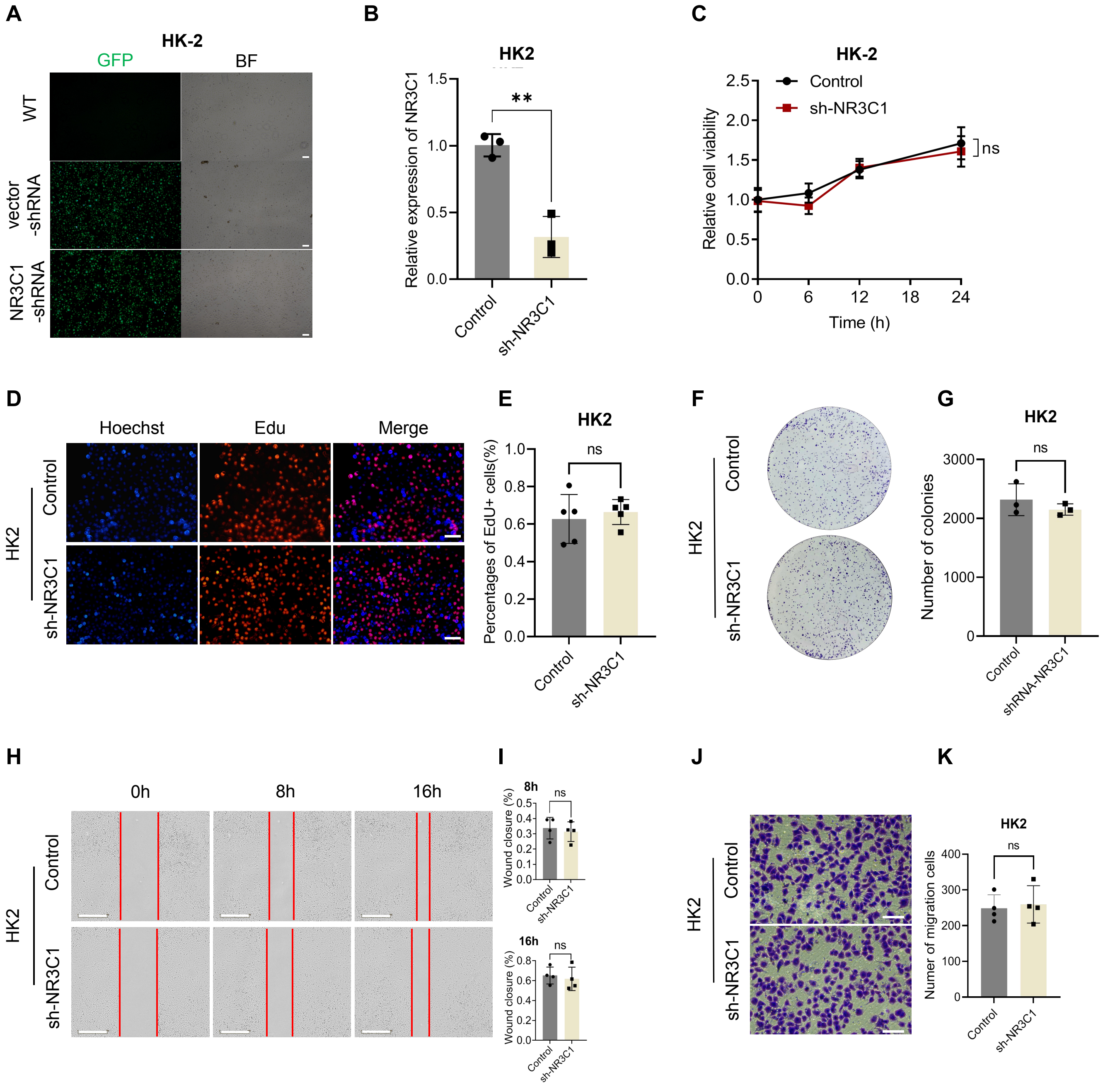

Supplement figure 4 Effect of NR3C1 knockdown on proliferation and migration of HK2 cells.

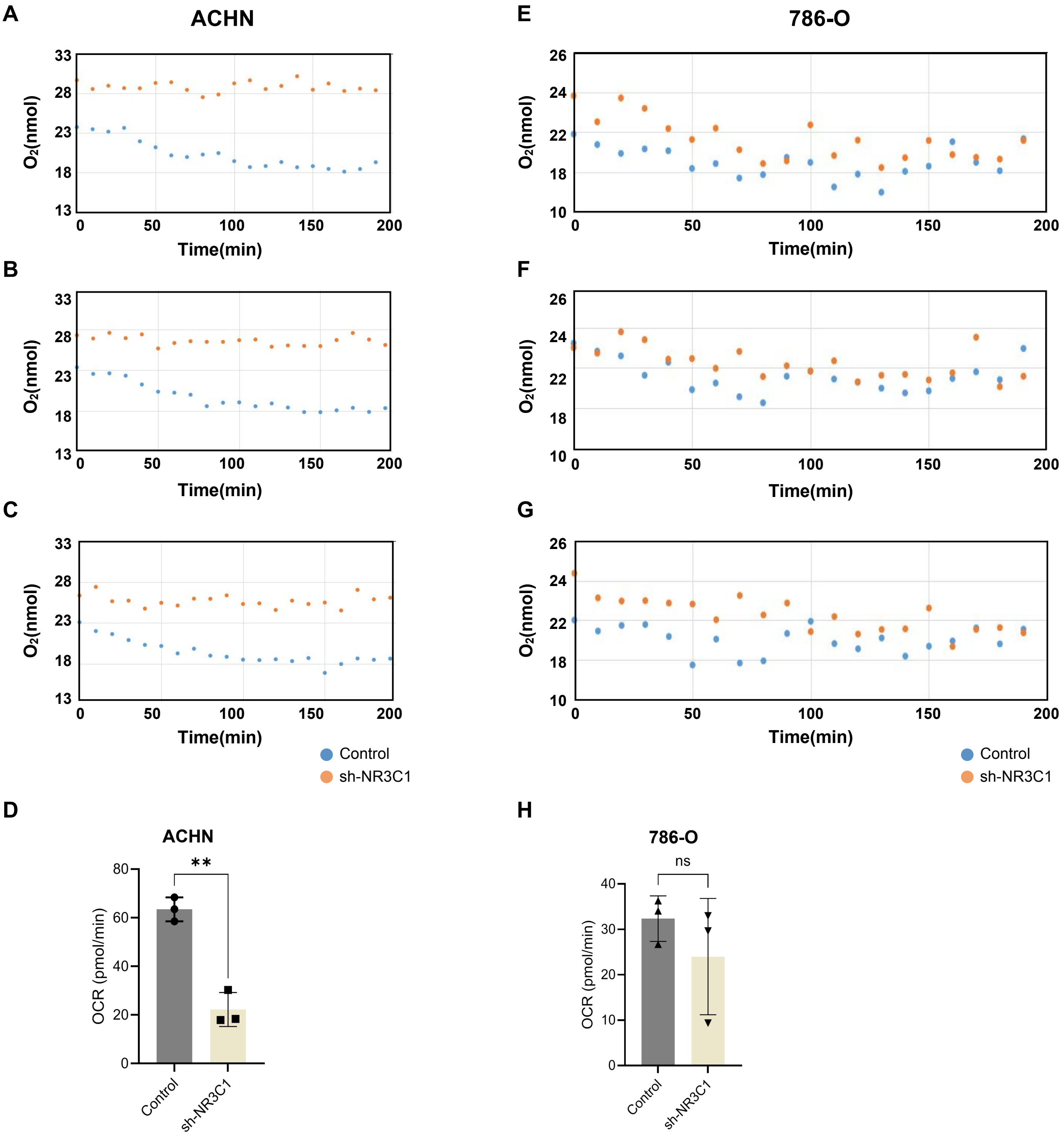

Supplement figure 5 The effect of NR3C1 knockdown on the oxygen consumption rate (OCR) in ccRCC cells.

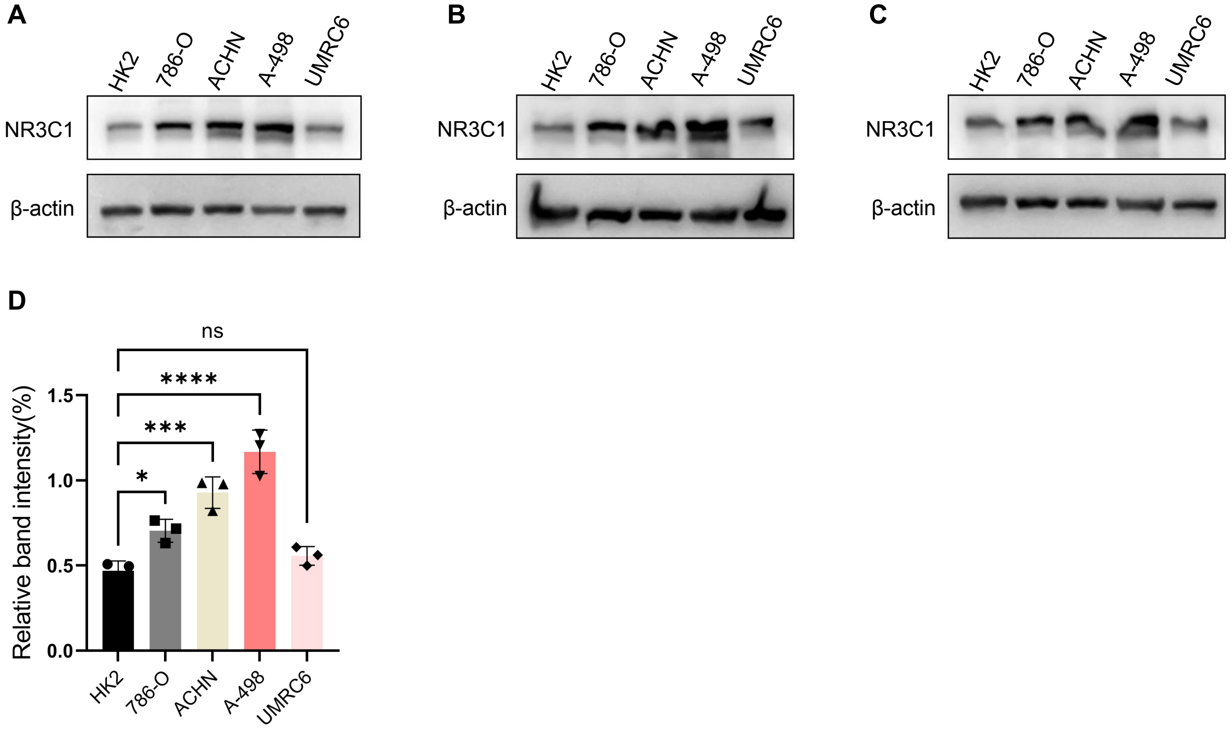

Supplement figure 6 Differences in NR3C1 expression between ccRCC cells and HK2 cells.
