# Supplementary material for: Knockdown of NR3C1 inhibits the proliferation and migration of clear cell renal cell carcinoma through activating endoplasmic reticulum stress–mitophagy
Source: J Transl Med. 2023 Oct 8;21:701. doi: 10.1186/s12967-023-04560-2 (PMC10560440; doi:10.1186/s12967-023-04560-2)
Supplement: Supplementary file 2 — Additional file 2: Table S1. Clinicopathological characteristics of ccRCC patients. [file 12967_2023_4560_MOESM2_ESM.docx]

| Clinicopathological characteristics | Details |
| --- | --- |
| Total number of patients | 10 |
| Age (median, range) | 67，56-74 |
| Gender (male/female) | 6/4 |
| Tumor side (left/right) | 5/5 |
| Surgery (radical nephrectomy/nephron sparing surgery) | 7/3 |
| T stage （1/2/3） | 5/1/4 |
| N stage （0/1） | 8/2 |
| M stage （0/1） | 8/2 |
| Pathological grade（1/2/3） | 3/3/4 |

Supplement table 1 Clinicopathological characteristics of ccRCC patients
